# PKAD: a database of experimentally measured pKa values of ionizable groups in proteins

**DOI:** 10.1093/database/baz024

**Published:** 2019-02-26

**Authors:** Swagata Pahari, Lexuan Sun, Emil Alexov

**Affiliations:** Computational Biophysics and Bioinformatics, Department of Physics and Astronomy, Clemson University, Clemson, South Carolina, USA

## Abstract

Ionizable residues play key roles in many biological phenomena including protein folding, enzyme catalysis and binding. We present PKAD, a database of experimentally measured pKas of protein residues reported in the literature or taken from existing databases. The database contains pKa data for 1350 residues in 157 wild-type proteins and for 232 residues in 45 mutant proteins. Most of these values are for Asp, Glu, His and Lys amino acids. The database is available as downloadable file as well as a web server (http://compbio.clemson.edu/pkad). The PKAD database can be used as a benchmarking source for development and improvement of pKa’s prediction methods. The web server provides additional information taken from the corresponding structures and amino acid sequences, which allows for easy search and grouping of the experimental pKas according to various biophysical characteristics, amino acid type and others.

## Introduction

Ionizable side chains in proteins play a key role in various functionalities of the corresponding proteins and protein complexes. The ionization state of titratable residues affects the function, stability, structure and solubility of protein (1–3). Ionizable residues also play a key role in protein folding (4, 5). pH dependence of protein stability and conformations can be explained in many cases by perturbed pKa values of ionizable residues (6–8). It is possible to identify the active site of the protein on the basis of predicting perturbed pKas (9, 10). Therefore, it is very important to understand and predict the pKa values of ionizable groups of proteins and which are the factors contributing to the corresponding pKa shifts.

The most widely used experimental method for determining the pKa of ionizable residues is the multidimensional nuclear magnetic resonance (NMR) spectroscopy (11–13). It has been found that NMR estimates the pKa values with the accuracy of 0.1 pK unit, making it the most precise method. Alternatively, pKa’s measurements have been performed using indirect techniques (14, 15) such as potentiometric titration, calorimetry, electrophoresis and high-performance liquid chromatography, typically resulting in larger error.

Although many experimentally measured pKa values are available in the literature, only few attempts have been made so far to compile experimental pKa values into a database. The availability of a large pKa’s data set for proteins with known 3D structures provides many opportunities. For example, it can be used for benchmarking the existing methods or establishing new one based on some training or empirical protocols. In this paper, we report the development of pKa’s database (PKAD). The PKAD database contains 1350 residue-specific pKa values for 157 wild-type proteins and 232 pKa values for 45 mutant proteins. The data were taken from literature and existing databases. In addition to providing experimentally measured pKas, the webserver offers the corresponding Protein Data Bank (PDB) ID, the error (uncertainty) in pKa measurements, salt concentration, pH range and temperature used for pKa measurement along with computed relative solvent accessible surface areas (%SASAs). Our goal is to provide a collection of pKa values along with corresponding 3D structures to assist the development and benchmarking of computational approaches to predict pKas. The database development and the details of the webserver tools usage are provided below.

## Database development

The web server back-end layer is implemented by a MariaDB relational database management system, which provides the infrastructure in archiving the structured pKa data, and a PHP server that communicates with the database to generate a cached JSON data file to reduce the system load. The front-end layer is built by HTML and JavaScript, which will load the JSON file, render the table and react to the user’s requests.

pKas of ionizable residues in wild-type proteins and mutant proteins are displayed in two different tabs in the web page. The web page will pull data from the database and shows up as a table with some searching fields. Users can type numbers or key words in these fields to filter the pKa range and to search on PDB ID, residue name or residue ID. The PDB ID link directs to the corresponding structure in PDB database (16). Users can also directly download the database.

## Database content

The PKAD database contains pKa values for 1350 ionizable residues from 157 wild-type proteins and 232 ionizable residues from 45 mutant proteins. They are collected from literatures and two other previously published databases: pKa cooperative (http://pkacoop.org/) (17) and Grimsley *et al.*’s data set (18). The pKa values are determined for ionizable side chains as well as C- and N-termini. Of the pKa entries in the database, 80% are for Glu, Asp, Lys and His residues. Therefore, most of our remarks will be on these groups. Very few data points are available for Tyr (47 cases), Cys (20 cases), C (23 cases) and N (21 cases) termini. Since the pKas of Ser and Arg residues are (>=12), it is difficult to measure the pKa of these residues by titration as protein denatures at such high pH, resulting in very few such experiments.

The database contains pKa values for both surface exposed and buried residues. We define relative %SASA as the percentage ratio of SASA in the protein versus free residue in water. The %SASA provided in the database can guide the user to distinguish between buried and surface exposed residues. Therefore, if one would like to focus on predicting pKa values of buried residues, an easily extractable subset of the database would serve as a benchmark. For each pKa, the database provides the ID of the corresponding 3D structure according to protein databank (PDB).

## Results and discussions

### Statistics of experimental pKas in wild-type proteins

**Figure 1:**
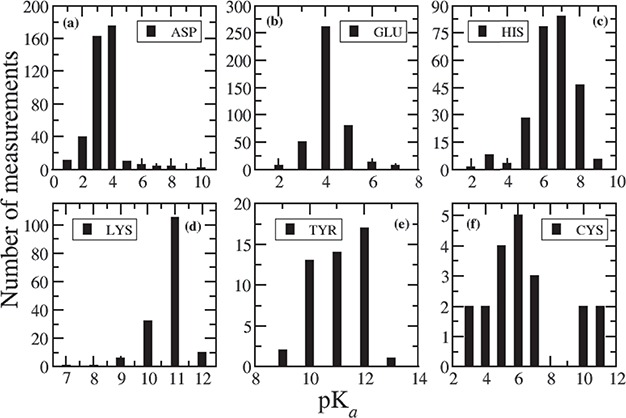
Distribution of measured pKa values for (**a**) ASP, (**b**) GLU, (**c**) HIS, (**d**) LYS, (**e**) TYR and (**f**) CYS.

A brief summary of measured pKa values for 1350 ionizable residues from 157 wild-type proteins is shown in Table 1. The summary is based on 408 Asp, 417 Glu, 253 His, 155 Lys, 47 Tyr, 20 Cys, 21 N-termini and 23 C-termini experimental pKas. The average, lowest and highest of the corresponding measured pKa values are also included in Table 1. The average pKa for Asp is 3.43, Glu = 4.14, His = 6.45, Lys = 10.68, Tyr = 10.98, Cys = 6.25, N-termini = 7.64 and C-termini = 3.16, which are very close to their intrinsic pKas. However, the measured pKa for each ionizable residue vary in a wide range of values depending on the surrounding environment, which is clearly seen in the corresponding distribution plots in Figure 1. For Asp, Figure 1a indicates that 92% of the pKa’s measurements fall between 2 and 4. The lowest value of 0.5 is observed for Asp76 in Ribonuclease T1 (RNase T1) and for Asp70 in T4 lysozyme. The side chain of Asp76 in RNase T1 is 99% buried, and it forms four hydrogen bonds with (i) a buried, conserved water molecule, (ii) Thr91, (iii) Tyr11 and (iv) Asn9 (14). These hydrogen bonds stabilize the aspartate and therefore drastically lower the pKa. In T4 lysozyme, the side chain of Asp70 makes salt bridge with His31, which stabilizes the protein by 3–5 kcal/mol (6) and is responsible for such low pKa (0.5). On the other hand, the highest pKa value of 9.9 is observed for Asp26 in reduced human thiredoxin (PDB ID: 1ERT) where the Asp26 is completely buried in the hydrophobic core of the protein and does not have any favorable interactions.

**Table 1:** Summary of experimentally measured 1350 residue-specific pKa values for wild-type proteins collected from literature

**Residue ID**	**No. of measurements**	**Average pK**_**a**_	**Lowest pK**_**a**_	**Highest pK**_**a**_
ASP	408	3.43	0.5	9.9
GLU	417	4.14	2.1	7.2
HIS	253	6.45	<2.3	9.19
LYS	155	10.68	6.5	12.12
TYR	47	10.98	6.08	12.5
CYS	20	6.25	2.88	11.1
C-term	23	3.16	2.4	4.03
N-term	21	7.64	6.91	9.14

For Glu, 93% of the pKa values are within 3 to 5, and 82% of the measured pKas are between 4 to 5 (Figure 1b). The lowest pKa of 2.1 is observed for Glu73 residue in barnase, where this residue is close to positively charged Arg83, Arg87 and Lys27 groups and acts as a general base in the catalytic site. Thus, the positively charged environment near Glu73 stabilizes the deprotonated form of Glu and lowers its pKa. The highest value for Glu is noticed as 7.2 for Glu172 in a family 11 glycosidase (PDB ID: 1HV0). Glu172 is buried inside the hydrophobic interior of the protein. The reason for elevation of pKa for Glu172 is that in addition to the desolvation penalty, there is an electrostatic repulsion with negatively charged Glu78, which is stabilized by strong hydrogen bond with Tyr69 and Gln127 (19).

In case of His, 82% of the measured pKa values are in the range between six and eight, and 93% fall between pKa values of 5 to 8 (Figure 1c). The lowest value of <2.3 is observed in case of His149 in Xylanases, thus His149 is never ionized in the folded protein. His149 is completely buried inside the hydrophobic core of the protein and participates in hydrogen bond interactions (20). Another low pKa value (2.5) is observed for His18 in horse cytochrome c. His18 forms two H-bonds with Ala15 and Pro30 (21), but it is coordinated to the iron of heme group, thus resulting in such unusually low pKa value. The highest value of 9.19 is observed for His72 in bovine heart phosphotyrosyl phosphatase (PDB ID: 1PNT). His72 is in close distance to two side chain carboxylates: Glu23 (3.3 Å) and Asp42 (4.3 Å) (22). Therefore, the main reason for elevation of pKa of His72 is the presence of negatively charged acidic residues.

Figure 1d indicates that 95% of the measured pKas for Lys residues are in the range between 10 and 12. Usually, Lys residues are more surface exposed than other charged residues (23). The lowest value of Lys is observed as 6.5 in a T4 lysozyme (PDB ID: 1L54), which is fully buried inside the hydrophobic core (24). The highest pKa value of 12.12 has been measured for Lys55 in bovine calbindin D9K. In this protein, Lys55 is situated close to negatively charged groups forming Ca binding site, which stabilize the ionized form of Lys55 and in turn cause a large shift in pKa (25).

There are limited number of pKa determined for Tyr (41), Cys (20), N- (14) and C-termini (15). In case of Tyr, 94% of the measured pKas are in the range of 10 to 12, and 98% are in the range of 10 to 13 (Figure 1e), whereas the average pKa is 10.99. The lowest pKa value of 6.08, which is approximately five units lower than the intrinsic pKa, is determined for Tyr149 in UDP-galactose 4-epimerase from *Escherichia coli* (26). This is due to the extra stabilization of the ionized form of Tyr149, caused by the positive electrostatic field generated by nearby NAD+ and Lys153. Additionally, Tyr149 forms hydrogen bond with Ser124. Tyr149 presents in catalytic site of the protein and provides the driving force along with Ser124 for mediating proton transfer (26). Tyr31 has the highest pKa value of 12.5 in third domain of silver pheasant ovomucoid (OMSVP3). Tyr31 is in close proximity to Asp27; therefore, this large shift in pKa is due to the charge–charge interaction between Tyr31 and the carboxylate (COO-) group of Asp27, which destabilizes the ionized form of Tyr31 (27).

In case of Cys, the average pKa (6.25) is lower than the intrinsic pKa of Cys. This is because most of the Cys residue entries in the database are situated in the catalytic site of the corresponding proteins. For example, in cysteine proteinases (PDB ID: 1PPO, 1PPN), Cys25-S-/His159-Imidazole(Im)+ ion pairs are common in the active site (28). The lowest measured pKa values for Cys are 2.88 in papaya protease (PDB ID: 1PPO) and 3.32 in papain (PDB ID: 1PPN). Therefore, we notice that pKa value of nucleophilic Cys is shifted downwards. This is because of the strong interaction with cationic histidine and, secondarily, from the N-termini of a α-helix (29). On the other hand, in a ubiquitin-conjugating enzyme (UBc13, PDB ID: 1JBB), the highest value of 11.1 is observed for Cys87 present in the active site of the enzyme. From the PDB structure, it is evident that Cys87 is surrounded by acidic residues, which prevent the deprotonation of Cys. This is the reason why we observe elevated pKa value (two pKa units larger than the intrinsic pKa) for this catalytic Cys.

### pKas and solvent accessible surface area

Solvation plays an important role in the ionization equilibria. Desolvation, due to moving an ionizable residue from solvent to protein, destabilizes the charged form of the residue, i.e. the extent of desolvation is more for charged compared to neutral form of the residues. Therefore, in absence of charge–charge and charge–dipole interactions, it is expected that acidic residues (Asp, Glu) while buried inside the protein, the corresponding pKas should be elevated. In contrast, buried basic groups are expected to have low pKa values. However, many studies in this regard show that the relationship between pKa and solvation is complex (30, 31). This is because of the presence of strong electrostatic interactions between the corresponding ionizable group and the rest of charges in the protein.

**Figure 2:**
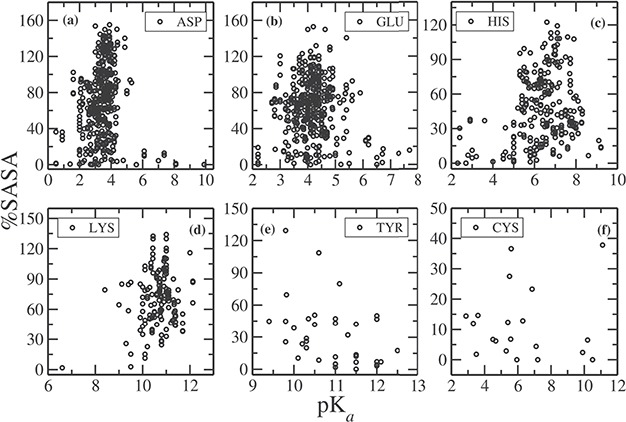
%SASA as a function of pKa for (**a**) ASP, (**b**) GLU, (**c**) HIS, (**d**) LYS, (**e**) TYR and (**f**) CYS.

In Figure 2, we plotted the relative %SASA of the side chains, calculated using NACCESS (32), as a function of pKa for each residue type (note that NACCESS uses standard value for SASA of a free residue, which in some cases results in %SASA larger than 100%). About 68% of Asp side chains have %SASA > 40 (Figure 2a) and the pKa values for these Asp residues are clustered narrowly around the mean pKa value of 3.4. For 30% of the Asp residues, %SASA < 40, and pKa values of these residues vary in a wide range, from 0.5 to 9.9. The residues, whose pKa values are perturbed to a large extent, are typically observed to be fully buried (%SASA < 10). For example, the pKa value of Asp102 in bovine chymotrypsinogenA (PDB ID: 1EX3) is 1.36, and its %SASA is 0.6; pKa of Asp76 in RNase T1 is 0.5, and its %SASA is 1.0; pKa of Asp26 in human thioredoxin (PDB ID: 1ERT) is 9.9, and the %SASA of the side chain is 0.2.

Similarly, for Glu, 75% of the residues have %SASA > 40 (Figure 2b), and the corresponding pKas vary in the range between 2.6 and 6 (the mean value is 4.15). There are Glu residues for which pKa value falls outside the range (<2.6, >6), and they have %SASA < 30, i.e completely or partially buried inside the protein. For example, pKa of Glu73 in barnase (PDB ID: 1A2P) is 2.1, and its %SASA is 8.3; pKa of Glu172 in family 11 glycosidase (PDB ID: 1HV0) is 7.2, and its %SASA is 15.8.

The pKa values for His residues having %SASA > 40, vary between 5.2 to 8 with a mean value of 6.4. On the other hand, all partially or fully buried His residues (having %SASA < 40) have lower (<5) or higher (>8) pKa values (Figure 2c). Similarly, for Lys residues, having %SASA > 40, pKa values are in between 9 and 12, around the mean value of 10.8. Only Lys55 in bovin calbindin D9K has pKa value of 12.12, and Lys39 in a mutt enzyme (PDB ID: 1MUT) is 8.4, which are slightly out of range, although the %SASA of the side chain is greater than 40. In case of Tyr, the pKas of all the residues having %SASA > 40 fall in the range between 9.4 and 12 (Figure 2e), centered around the mean value of 10.8. There are few Tyr residues, which have pKa values of >12.0. The side chain of all these residues have %SASA < 20. Most of the Cys residues in our data set are placed in the catalytic site of the protein, thus are fully or partially buried and having %SASA < 30 (Figure 2f). The average pKa of all the Cys residue entries is 6.25. The residues having larger pKas (>7) have %SASA < 10, and those having smaller pKa (<5) have %SASA < 15. Therefore, side chain of these residues is completely buried in the hydrophobic core of protein. The only exception is Cys87 in a ubiquitin-conjugating enzyme (UBc13, PDB ID: 1JBB), whose pKa is 11.1, although having %SASA of 37.8.

### Statistics of pKas in mutant proteins

Two types of mutations are presented in PKAD: (i) mutations that do not involve the residue for which pKa is considered and (ii) mutations that introduce a new titratable group. In the first case, mutation affects the pKa of an existing titratable group by either altering the accessibility or interactions, and therefore, such cases will be referred in this work as mutant type I (Mut-I). In the second case, the mutation is a placement of a new titratable group and will be referred as Mut-II.

**Figure 3:**
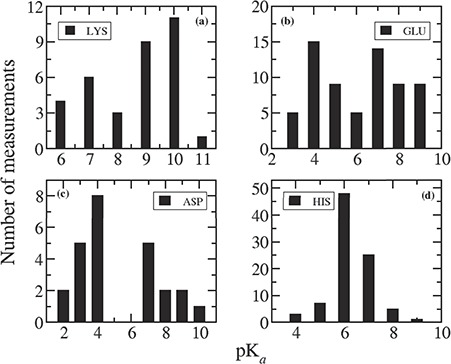
Distribution of measured pKa values for (**a**) LYS, (**b**) GLU, (**c**) ASP and (**d**) HIS.

We plotted the distribution of measured pKas for individual residues in mutant proteins in Figure 3. It can be noticed comparing Figure 3 with Figure 1 that the pKa values in mutant proteins are quite different compared to that in wild-type proteins. Indeed, in mutant proteins, the pKas are much more shifted away from the corresponding intrinsic values.

For most of the Lys residues (28 out of 35 entries) present in the database, mutations are Mut-II, and this is why the pKa values are so different than in wild type. The average pKa of Lys is 8.54, which is 2.14 pK unit lower than the average pKa found in wild-type protein (10.68). Figure 3a indicates that the pKa values for Lys can vary from 5 to 11 with a significant probability at 6 and 7. Probability of having pKa = 10 is the highest and pKa = 9 is slightly lower. In case of wild-type proteins, pKas of Lys mostly lies between 9 and 12.

There are 66 pKa values for Glu residues reported in the database for mutant proteins. Among them, 36 are Mut-II cases, and the remaining 30 are Mut-I. Therefore, one can see a wide distribution of pKa values for Glu in the range between 2 and 9. The elevated pKa values of Glu are mostly observed for Mut-II cases because the newly placed residue is in non-native environment, and thus non-native charge–charge and charge–dipole interactions. For the rest of the cases (Mut-I class), the pKas are quite similar to that in wild-type proteins.

Similarly, among 25 Asp residues in the mutant database, 9 of them are Mut-II class, and their pKas are significantly perturbed. In the remaining 16 cases (Mut-I class), the pKas are close to that in wild-type protein.

For His residues, all the 89 entries belong to Mut-I class. Therefore, the average pKa (6.33) of all the His residues in mutant database is similar to that (6.45) in wild-type cases. Figure 3d indicates that pKas of His residues vary in the range of 4 to 9, and 82% of them lie between 6 and 7.

There are very few entries for Cys residues in the mutant database, thus it is difficult to comment on their pKas. However, there is significant perturbation for most of the Cys pKa values. This is because most of the Cys residues, present in the mutant database, are placed in the catalytic site of the protein.

## Conclusions

PKAD database is developed to provide an easily accessible collection of experimentally measured pKa values. The database can be used in many different applications throughout different disciplines. The easy downloadable, searchable option, along with a large number of entries, makes the PKAD database unique. The size of the database will increase as more experimentally measured pKa values will be published. The database is accessible via http://compbio.clemson.edu/pkad.

It should be mentioned that in some cases (strongly coupled titratable groups), the titration behavior may be quite complicated resulting in a titration curve that does not follow Henderson–Hasselbalch equation. Such cases cannot be described by a single pKa value; instead, one should model the titration curve and compare it with experimental data (33). A database of experimentally measured titration curves was reported in reference (34) and can be used for such purpose.

## Notes

**Database URL**: http://compbio.clemson.edu/pkad
